# Psoas muscle abscess as initial manifestation of disseminated tuberculosis in a previously healthy man: a case report

**DOI:** 10.1590/S1678-9946202567025

**Published:** 2025-04-14

**Authors:** Pedro Henrique Moreira Barbosa, Ezequias Batista Martins, Billy McBenedict, Remberto Maurício de La Cruz Vargas Vilte, Karla Regina Oliveira de Moura Ronchini, Natalia Chilinque Zambão da Silva, Patrícia Yvonne Maciel Pinheiro, Thais de Oliveira Vieira, Bianca Balzano de la Fuente Villar

**Affiliations:** 1 Hospital Universitário Antônio Pedro, Departamento de Infectologia, Niterói, Rio de Janeiro, Brazil; 2 Universidade Federal Fluminense, Faculdade de Medicina, Niterói, Rio de Janeiro, Brazil

**Keywords:** Tuberculosis, Mycobacterium tuberculosis, Psoas abscess, Spondylodiscitis

## Abstract

Psoas muscle abscess is an insidious disease, with varied clinical manifestations and a challenging diagnosis. This pathology has been more frequently identified due to the increased availability of high-quality radiological imaging, such as computed tomography. In Brazil, *Mycobacterium tuberculosis* is the most common secondary etiologic agent of psoas abscess. We report the case of a 28-year-old immunocompetent man diagnosed with disseminated tuberculosis, affecting the lungs, lumbar spine, and psoas muscle, leading to permanent locomotion sequelae. This case is very relevant for osteoarticular complaints, as low back pain and limping were the initial symptoms. Diagnosis was confirmed by ultrasound-guided percutaneous drainage of the psoas muscle abscess and detection of the *M. tuberculosis* complex via Xpert MTB/RIF. A 12-month treatment with antitubercular drugs was effective.

## INTRODUCTION

Psoas muscle abscess is a rare disease that primarily affects young adults and men. In 1992, its annual global incidence was estimated at 12 cases/year, but current incidence is unknown^1^. Psoas abscess can be classified as primary or secondary, depending on the presence of infection in adjacent organs. Primary psoas abscess typically occurs due to hematogenous infection spread and is often seen in immunocompromised individuals, without direct involvement of nearby organs^1^. Secondary psoas abscess results from an infection in adjacent structures, such as the gastrointestinal tract, the genitourinary system, sacroiliac joint or the spine. Symptoms tend to be varied and non-specific, with the most common being fever, back pain, and functional limitation of the lower limbs^2^.

In tropical regions with a high prevalence of tuberculosis (TB), such as Asia, Africa and South America, *Mycobacterium tuberculosis* is a significant cause of psoas abscesses^3^. Osteoarticular TB represents approximately 1% to 3% of all TB cases, primarily affecting the spine and weight-bearing joints^4^. This article reports a compelling case of disseminated TB in a previously healthy 28-year-old man, involving the psoas muscle, lumbar spine, and lungs.

### Ethics

Informed consent was obtained from participants involved in the study. The local ethics committee reviewed and approved the study and assigned the approval N° CAAE: 67709423.6.0000.5243.

## CASE REPORT

A previously healthy 28-year-old man presented to an outpatient clinic with a history of progressive low back pain radiating to the right lower limb, accompanied by a burning sensation for over a year. The pain worsened with walking and was alleviated by common analgesics. There was no history of trauma, fever, weight loss, respiratory symptoms, or skin changes. Physical examination revealed a decreased range of motion in the right hip joint, with limitations in abduction, flexion, and extension, as well as pain upon abduction of the right hip.

**Figure 1:**
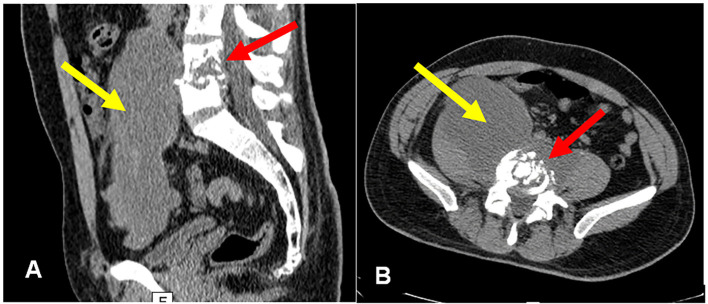
Abdominal MRI showing: (A) Spondylodiscitis involving fourth and fifth lumbar vertebrae (red arrow); (B) Bulky abscess of the right ileum psoas muscle (yellow arrow).

Abdominal and pelvic magnetic resonance imaging (MRI), performed on April 16, 2024, revealed an infiltrative lesion in the fourth and fifth lumbar vertebrae, with intermediate collections and diffuse contrast restriction. The lesion destroyed the adjacent vertebral plateaus and extended into the paravertebral space and posterior longitudinal ligament. A large, elongated abscess was identified in the right psoas muscle (Figure 1). The initial diagnosis was a psoas abscess with spondylodiscitis of undetermined etiology. Due to the lack of a definitive diagnosis, no specific treatment was performed. The patient was medicated only with potent analgesia (codeine 30mg, four times per day).

On May 14, 2024, 12 months after onset of symptoms, the patient was admitted to a university hospital for diagnostic evaluation and treatment. Vital signs were as follows: temperature of 36.7°C; blood pressure of 148/90 mmHg; a heart rate of 92 bpm, and a respiratory rate of 18 breaths per minute with oxygen saturation above 98% on ambient air. Laboratory tests were then conducted, including serological, biochemical, and hematological assessments. Serological tests for human immunodeficiency virus, syphilis, and viral hepatitis were negative, and hematological and biochemical parameters showed no abnormalities.

**Figure 2:**
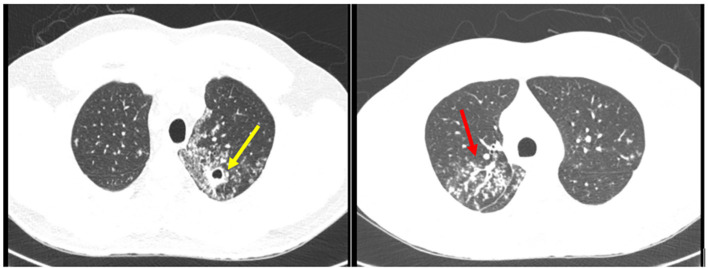
Chest CT showing right lung apex with multiple central lobular nodules, in a "tree-in-bud" pattern (red arrow). Cavitated lesion with thickened walls with 1.0 cm in diameter (yellow arrow).

Chest computed tomography (CT) on May 18, 2024, revealed multiple small centrilobular nodules within the lung parenchyma, with branching ground-glass opacities displaying a tree-in-bud pattern in both lungs. Thick-walled cavitary lesions, approximately 1 cm in diameter, were noted at the right lung apex (Figure 2). Bronchoscopy, performed the same day, revealed TB-suggestive lesions . Bronchoalveolar lavage (BAL) analysis confirmed the presence of *M. tuberculosis* complex, with no rifampicin resistance detected by the Xpert MTB/RIF^®^ assay, and smear microscopy for acid-fast bacilli was positive.

On May 19, 2024, five days after hospitalization, the patient underwent ultrasound-guided percutaneous drainage of the right psoas muscle abscess, yielding purulent material. The sample was sent for direct microscopy and cultures for common bacteria, fungi, and mycobacteria. The diagnosis was confirmed by *M. tuberculosis* complex detection via Xpert MTB/RIF^®^, with no rifampicin resistance. Cultures showed no growth of other organisms, and the acid-fast bacilli test was negative. The patient was started on a two-month regimen of rifampicin (750 mg/day), isoniazid (375 mg/day), pyrazinamide (2000 mg/day), and ethambutol (1375 mg/day) for TB (the first two months with the four drugs, followed by rifampicin and isoniazid for 10 months).

On May 23, 2024, ten days after hospitalization, the patient was discharged. Since May 20, 2024, he has been receiving outpatient follow-up in infectious disease and neurosurgery. He continues to experience low back pain that remains refractory to conventional analgesics. Due to severe neuropathic pain in the lower back, he was prescribed high doses of gabapentin (1800 mg/day) and carbamazepine (600 mg/day). Destruction of the fourth lumbar vertebra causes spinal compression, contributing to his pain. The patient developed chronic pain that has been challenging to manage and is exacerbated by movement. A noticeable limp is present when he walks. He is currently receiving conservative treatment for the vertebral fracture under the care of the surgical team, and corpectomy is being considered.

## DISCUSSION

Disseminated TB is characterized by the involvement of two or more non-contiguous sites due to hematogenous spread of the bacteria, resulting from primary infection, reactivation of a latent focus, or of iatrogenic origin^5^. This case describes an uncommon progression of TB in a young, immunocompetent patient, with dissemination to the lumbar spine and psoas muscle, without respiratory symptoms or signs of systemic compromise. The initial presentation of osteoarticular symptoms posed a diagnostic challenge, as they were the first signs of the disease.

The classic presentation of TB includes fever, back pain, and limping, though these findings may not always be present. Given the rarity of this disorder, evidence on its etiology, natural history, ethnic variations, and outcomes is limited^6^. Spinal TB typically presents with spine pain, tenderness, neurological deficits, cold abscesses, and fever. In advanced cases, it can result in kyphotic deformities and spinal instability. However, clinical presentation depends on the duration of the disease, the severity of the spinal destruction and the location of the infection^7^. The only symptom in our patient was low back pain, with no pulmonary or systemic symptoms, which delayed diagnosis.

The most common cause of primary psoas abscess is Gram-positive bacteria, with *Staphylococcus aureus* responsible for most cases^8^. Secondary psoas abscesses typically result from the local infection spread from an adjacent focus, with the most common sources being peritoneal pathologies and spinal infections. In developing countries, spinal TB (known as Pott's disease) is the leading cause of secondary psoas abscess^9^. In the presented case, the spread of *M. tuberculosis* to the psoas muscle could have occurred by two possible routes: direct extension from an infectious focus in the lumbar spine or hematogenous dissemination. Most likely, the spread occurred due to proximity to the lumbar spine, given the advanced destruction of the lumbar vertebrae.

Mycobacterial culture remains the gold standard for laboratory TB diagnosis. To overcome microscopy limitations and enable the recovery of mycobacteria for drug susceptibility testing, mycobacterial culture facilities have been established in resource-limited settings. Egg-based media, such as Lowenstein–Jensen (LJ), were among the first introduced due to their availability, ease of preparation, low cost, and ability to support small numbers of bacilli growth while inhibiting contaminants^10,11^. However, due to the fastidious and slow-growing nature of *Mycobacterium tuberculosis*, growth on solid media can take several weeks^11^. Although culture testing is highly recommended, in this case, cultures were negative and did not help clarify the diagnosis.

Studies indicate the Xpert MTB/RIF^®^ assay has a sensitivity ranging from 61.8% to 85.0% and specificity of 98% to 99% in detecting pulmonary TB from respiratory secretion samples^12^. However, it has been shown that the assay's sensitivity decreases in samples with a low bacterial load, limiting its effectiveness in identifying patients with negative sputum smear microscopy and in detecting extrapulmonary TB^13^. Despite this limitation, the positive test result from the abscess sample in this case suggests the assay can be useful to diagnose extrapulmonary TB.

The mortality rate can reach up to 2.4% for primary psoas abscess and 18.9% for secondary psoas abscess^14^. Extended treatment may be warranted in cases of HIV coinfection or non-drainable abscesses^15^. Studies suggested extending treatment to 24 months for selected cases, particularly those with multidrug resistance^16^. Our patient responded well to treatment, which included percutaneous abscess drainage and anti-TB medications. However, vertebral fractures often require surgical correction, which may lead to permanent osteoarticular sequelae. Furthermore, chronic neuropathic pain is a common persistent symptom.

Diagnosing lumbar spine infections and psoas abscesses can be challenging and often delayed due to their non-specific symptoms. A high level of suspicion is essential for early diagnosis, particularly in older patients with vague symptoms. In individuals with signs of infection, back or hip pain, or history of spinal surgery, psoas abscess should always be considered. Early diagnosis is crucial, as delayed treatment can lead to poor outcomes and serious health complications, as demonstrated in this case^17^.

## CONCLUSIONS

Diagnosing and managing psoas muscle abscesses can be especially challenging when they present initial manifestations of disseminated TB. Identifying *M. tuberculosis* as the causative agent in cases of psoas abscess and spondylodiscitis highlights the need for a high level of suspicion in regions with high TB prevalence. Recognizing TB as a potential cause of psoas abscess can facilitate earlier and more effective treatment. This case underscores the importance of considering differential diagnoses and conducting appropriate imaging studies when evaluating persistent low back pain, particularly in endemic regions.
